# Multilevel Differential Control of Hormone Gene Expression Programs by hnRNP L and LL in Pituitary Cells

**DOI:** 10.1128/MCB.00651-17

**Published:** 2018-05-29

**Authors:** Lei Lei, Wenguang Cao, Ling Liu, Urmi Das, Yujia Wu, Guodong Liu, Muhammad Sohail, Yangjun Chen, Jiuyong Xie

**Affiliations:** a Department of Physiology & Pathophysiology, Max Rady College of Medicine, Rady Faculty of Health Sciences, University of Manitoba, Winnipeg, Manitoba, Canada; b Department of Applied Computer Science, University of Winnipeg, Winnipeg, Manitoba, Canada

**Keywords:** hnRNP paralogs, divergence, prolactin, growth hormone, RNA-Seq, cryptic splicing, intron, alternative splicing, hnRNP

## Abstract

The pituitary-derived somatolactotrophe GH_3_ cells secrete both growth hormone (GH) and prolactin (PRL). We have found that the hnRNP L and L-like (LL) paralogs differentially regulate alternative splicing of genes in these cells. Here, we show that hnRNP L is essential for PRL only, but LL is essential for both PRL and GH production. Transcriptome-wide RNA sequencing (RNA-Seq) analysis indicates that they differentially control groups of hormone or hormone-related genes involved in hormone production/regulation at total transcript and alternative exon levels. Interestingly, hnRNP L also specifically binds and prevents the aberrant usage of a nonconserved CA-rich intron piece of *Prl* pre-mRNA transcripts, and many others involved in endocrine functions, to prevent mostly cryptic last exons and mRNA truncation. Essential for the full hnRNP L effect on specific exons is a proline-rich region that emerged during evolution in vertebrate hnRNP L only but not LL. Together, our data demonstrate that the hnRNP L and its paralog, LL, differentially control hormone gene expression programs at multiple levels, and hnRNP L in particular is critical for protecting the transcriptome from aberrant usage of intronic sequences. The multilevel differential control by hnRNPs likely tailors the transcriptome to help refine and safeguard the different gene expression programs for different hormones.

## INTRODUCTION

Differentiated cells maintain specific programs of precise gene expression for physiological functions. Perturbation of functions such as hormone production or response would have serious consequences. Of the many factors involved, the heterogeneous ribonucleoproteins (hnRNPs) are a family of abundant and critical players in the posttranscriptional processing of pre-mRNA transcripts (1, 2). They have evolved to about 30 members, including homologs or paralogs (3), for the tens of thousands of gene transcripts in vertebrates. This number is many more than those in fly, nematode, plant, or yeast (3). However, the role of the different, particularly paralogous, vertebrate hnRNPs in relation to specific cell functions and the accompanying gene expression programs remains largely unclear.

Among their diverse functions, gene regulation at the transcript and exon levels in vertebrate, particularly mammalian, systems has been well known (1, 3–5). Relatively less explored is the protection of transcriptome integrity from cryptic sites in the expanded introns, which harbor numerous signals for cryptic splicing and pieces that could be aberrantly processed into the mRNA under abnormal or disease conditions (3, 6). It has been shown that the exonization of Alu repeats can be prevented by hnRNP C (7), but it is not clear whether its diverged paralog, CL1, has a similar or distinct role in the process. A similar repression effect on cryptic exons has also been found in the cases of polypyrimidine tract-binding protein 1 (PTBP1; hnRNP I) and PTBP2 as well as TDP-43 (8, 9). The role of the many other hnRNPs, particularly the highly similar paralogs in the transcriptome-wide inhibition of cryptic splicing in relation to cell function, remains unclear.

hnRNP paralogs share high levels of sequence similarities but also diverged specific properties. For example, hnRNP A1/A2 or F/H have partial or nearly complete complementary effects on splicing in RNA interference assays of some exons (10, 11). PTBP1 and PTBP2 are both splicing repressors but have differential activities and switch expression for specific splicing events during neuronal differentiation (12, 13). hnRNP E1, but not E2, reduces the expression of the *Rev* gene of HIV-1 due to their different C termini (14). Therefore, elucidating distinct properties and transcript targets of hnRNP paralogs will help reveal the logic of their expansion and orchestration of the processing of particular mRNA targets in cell functions/diseases.

hnRNP L and L-like (LL) are paralogs that likely emerged in vertebrates through gene duplication (3, 15). They share approximately 55% overall identity between their primary amino acid sequences in humans and rats. They both prefer to bind CA/AC-rich motifs (16, 17) and play important and sometimes distinct roles in the normal processes of gene regulation and cell differentiation (18–26). In particular, hnRNP L preferentially binds to introns in the transcriptome and is thought to regulate alternative splicing (27). Moreover, an analysis of microarray data confirmed 11 exons that appeared to be specifically controlled by hnRNP L but not LL (21). However, comparison data between the paralogs using larger-scale techniques, such as transcriptome-wide RNA sequencing (RNA-Seq), and their effects on cryptic exons and cell functions have not been reported.

We have found that hnRNP L and LL differentially regulate the alternative splicing of genes involved in hormone production in GH_3_ rat pituitary cells (28–30). These clonal cells produce two structurally related hormones, prolactin (PRL) and growth hormone (GH) (31). Inside the pituitary, these hormones are produced from the *Prl* and *Gh1* genes in distinct lactotrophes and somatotrophes differentiated from common precursors (32). Deregulation of these genes in humans causes dwarfism (33), infertility, or puerperal alactogenesis (34–36). The expression of both hormones in the GH_3_ somatolactotrophes thus provides an ideal system for us to compare the global effects of the diverged hnRNP paralogs on the transcriptome and their potential impact on two distinct cell functions.

In this report, we show that the diverged hnRNP L and LL paralogs differentially control the production of PRL and GH hormones, as well as the transcript and alternative exon levels of many other genes involved in endocrine functions. Moreover, hnRNP L not only regulates gene expression but also prevents aberrant splicing of *Prl* and many other transcripts in the transcriptome, distinctively from its paralog, LL. We further show that this paralog-specific effect to protect transcripts requires an evolutionarily diverged domain of vertebrate hnRNP L for specific exons.

## RESULTS

### hnRNP L and LL differentially control the production of PRL and GH hormones.

**FIG 1:**
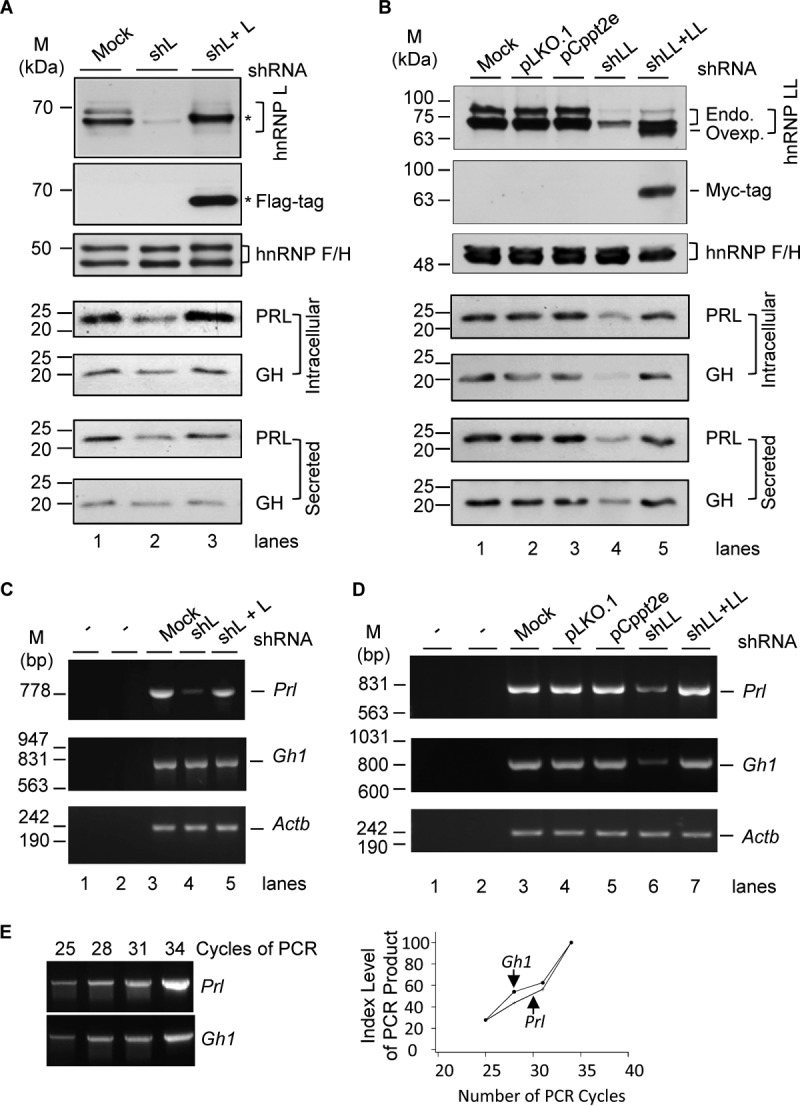
Control of GH and PRL hormones and transcripts by hnRNP L and hnRNP LL in GH_3_ pituitary cells. (A and B) Representative Western blots of the cellular and secreted prolactin and growth hormone of the GH_3_ cells with or without hnRNP L or LL knockdown (shL or shLL)/rescue (shL + L or shLL + LL), respectively, with hnRNP F/H as a loading control. pLKO.1 and pCppt2E, vector controls for shLL and Myc-hnRNP LL, respectively. *, Flag-tagged hnRNP L. In panel B, endogenous (Endo.) and overexpressed (Ovexp.) hnRNP LL are indicated to the right of the gel. (C and D) Agarose gels of the RT-PCR products of *Gh1* and *Prl* in the GH_3_ cells shown in panels A and B. −, PCR or RT negative control; *Actb*, RNA loading control. (E) PCR linearity test by cycle numbers for the *Prl* and *Gh1* genes. Agarose gels of the products from PCR cycles 25 to 34 and plots of the product intensity versus cycle numbers are shown.

Our previous observation of hnRNP L/LL regulation of alternative splicing of hormone-related genes such as the *Slo1* potassium channel gene suggests that they control hormone production (28–30). We thus examined PRL and GH in the cellular protein lysates or culture media of the hnRNP L- or LL-knockdown samples by Western blotting (Fig. 1). Upon hnRNP L knockdown by expressed lentiviral short hairpin L (shL) RNA (28, 29), the protein levels of both intracellular and secreted PRL were reduced by about 60% (*P* value of <0.001; *n* ≥ 6), but those of GH were not changed significantly. Importantly, the PRL level was nearly fully recovered by lentiviral hnRNP L-Flag expression (*n* ≥ 3) (Fig. 1A). In contrast, upon hnRNP LL knockdown by lentiviral shLL RNA (29), both the intracellular and secreted PRL and GH were reduced by about 80% (Fig. 1B) (*P* value of <0.001; *n* = 3). The reduction was prevented by lentiviral Myc-hnRNP LL expression. Together, these data indicate that the hnRNP paralogs have distinct roles in the production of the two hormones: hnRNP L controls PRL only, while hnRNP LL controls both.

We then examined the corresponding hormone transcripts *Prl* and *Gh1* in these cells by semiquantitative reverse transcription-PCR (RT-PCR). The result confirmed the same trends of changes of both transcripts (Fig. 1C to E): hnRNP L controls *Prl* only, but hnRNP LL controls both *Prl* and *Gh1*.

### hnRNP L and LL differentially control hormone gene expression programs at both total transcript and alternative exon levels.

**FIG 2:**
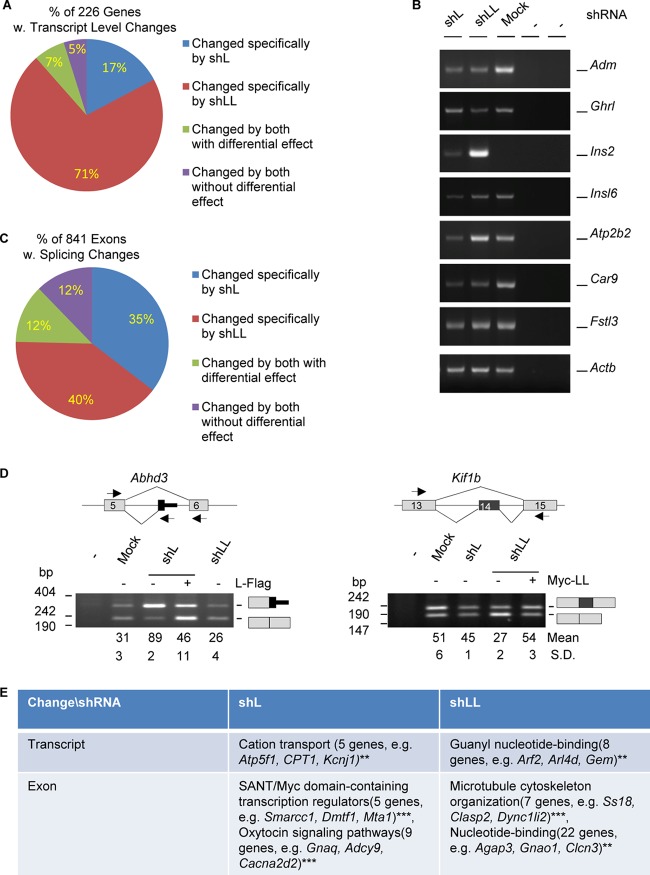
Profiles of transcripts and alternative exons controlled by hnRNP L and LL in GH_3_ cells. (A) Pie-shaped percent distribution of genes with fold changes of less than 0.5 or more than 2.0 (*P* < 0.05) and average read numbers of at least 50. (B) Representative agarose gels of RT-PCR products of a group of hormone or hormone-related genes differentially expressed in the knockdown samples. Adm, adrenomedullin; Ghrl, ghrelin; Ins2 e3, insulin 2, exon 3; Insl6, insulin-like 6; Atp2b2, ATPase, Ca^2+^ transporting, plasma membrane 2; Car9, carbonic anhydrase 9; Fstl3, follistatin like 3; Actb (beta-actin), RNA loading control; −, PCR or RT negative control. (C) Pie-shaped percent distribution of alternative exons changed by shL, shLL, or both. (D) Examples of alternative exons specifically changed by shL or shLL. Lines, introns; gray boxes, constitutive exons; black boxes, alternative exons; arrows, PCR primers; Abhd3, abhydrolase domain containing 3; Kif1b, kinesin family member 1b. (E) DAVID function clustering analysis of the most significantly clustered functions of genes changed at transcript or exon levels specifically by shL or shLL. **, *P* < 0.01; ***, *P* < 0.001.

To compare the transcriptome-wide effect of hnRNP L and LL in their differential control of hormone production, we carried out RNA-Seq analysis (see Materials and Methods for more details) among three groups of GH_3_ samples (mock, shL, and shLL). By edgeR analysis of the changes of total transcript levels, we identified 412 shL- and 984 shLL-specific genes that showed less than 0.67 (decreased)- or more than 1.5 (increased)-fold difference in the knockdown over the mock group. Of these genes, only 160 were found in both groups. We further narrowed down the list to 226 genes with less than 0.5- or more than 2-fold difference. Most (88%) of these genes were specific for either shL (17%) or shLL (71%), with the remaining 12% changed by both (Fig. 2; see also Table S1 in the supplemental material). Moreover, most (83%) of the 226 genes were changed by shLL, and 83% of these shLL targets (133 genes) were reduced, consistent with a prominent role of hnRNP LL in stabilizing mRNA transcripts in another study (19). Similarly, most of the shL targets (75%) were reduced as well. By semiquantitative RT-PCR, we successfully validated the changes of 25 out of 28 genes examined (Fig. 2B), supporting the accurate prediction by the edgeR analysis. These include the adrenalmedullin (*Adm*), ghrelin (*Ghrl*), carbon anhydrase 9 (*Car9*), and insulin 2 (*Ins2*) genes, among others.

We also analyzed the changes of alternative exons using DEXSeq (37). We identified 841 exons/regions (bins) that changed significantly between at least two pairs of the three groups of samples (≥1.1-fold; *P* value of <0.05) (Fig. 2C and D and Table S2). The majority (75%) of the exons were changed specifically by shL (35%) and shLL (40%). We manually examined the exons in the Integrative Genomics Viewer (IGV) and verified 31 of 33 alternative splicing events by RT-PCR (94%) (Fig. 2D) (unpublished data), supporting the accurate prediction of regulated exons by our DEXSeq analysis. Of the 453 Ensembl genes of the changed exons/bins, only 6 (∼1.3%) were also changed at the transcript level in Fig. 2A, suggesting that the splicing control by hnRNP L or LL is mostly independent of its regulation of transcript levels.

Functional analysis indicates that these shL- or shLL-specifically changed genes cluster for different aspects of endocrine functions at both the total transcript and exon levels (Fig. 2E). Specifically, genes changed at the transcript level by shL cluster for cation transport but those changed by shLL cluster for guanyl nucleotide binding, while those changed at the exon level by shL cluster for transcription control and oxytocin signaling but those changed at the exon level by shLL cluster for microtubule organization and nucleotide binding. These functions are related to cellular excitability, signaling, cytoskeleton rearrangement, hormone vesicle transport/release, and gene transcription, processes that are important in different steps of hormone production and regulation.

### hnRNP L also specifically inhibits a cryptic exon of *Prl* by binding to intronic CA-rich motifs.

**FIG 3:**
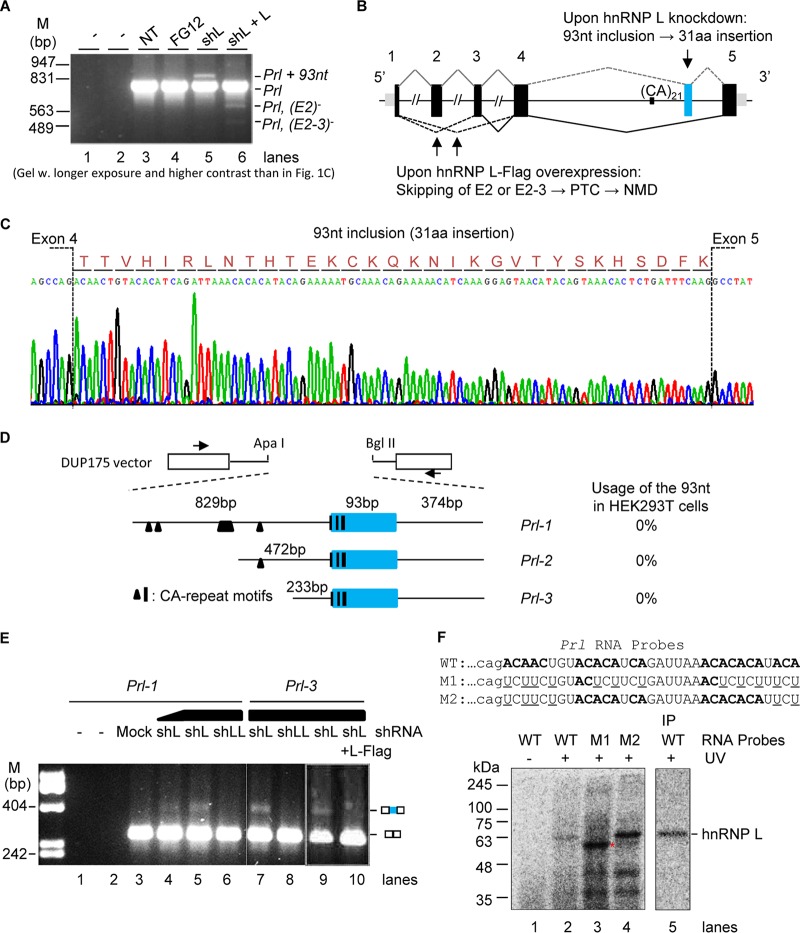
hnRNP L-specific effect on the usage of a 93-nt cryptic exon of *Prl*. (A) A high-contrast, long-exposure image of an agarose gel of the RT-PCR products of *Prl* in GH_3_ cells. Identities of the different *Prl* products are to the right of the gel. Note that this gel highlights the longer Prl product and is not quantitative regarding the Prl level compared to that depicted in Fig. 1. (B) Diagram of the *Prl* variants detected in GH_3_ cells with different expression levels of hnRNP L. Lines, introns; boxes, exons; narrower boxes, untranslated regions; blue box, the intron piece (cryptic exon) retained in the shL sample. (C) Sequencing chromatogram of the Prl + 93-nt band in the gel shown in panel A. Inclusion of a 93-nt cryptic exon between exons 4 and 5 of *Prl* caused a 31-aa (amino acid) insertion in the PRL protein. (D) Diagram of the *Prl* splicing reporter minigenes cloned into the vector DUP175. Black bars and triangles, CA-rich motifs; horizontal lines, introns; blue box, the 93-nt cryptic exon. Test results of the 93-nt usage in the HEK293T cells are on the right. Arrowheads indicate the location of PCR primers. (E) hnRNP L specifically inhibits splicing of the 93-nt cryptic exon. Shown is an agarose gel of the RT-PCR products of *Prl* minigenes from HEK293T cells with or without hnRNP L or LL knockdown and rescue. Relative amounts of the shRNA viruses used for transduction are 30 μl and 90 μl. Identities of the PCR bands are to the right. (F) UV cross-linking assay of the [α-^32^P]CTP-labeled *Prl* RNA probes in HeLa nuclear extracts, followed by immunoprecipitation (IP) with anti-hnRNPL. (Top) CA-rich motifs of the *Prl* RNA probes. (Bottom) Phosphor images of the cross-linked and IP proteins in SDS-PAGE gels. *, uncharacterized protein, likely PTBP1, with increased binding to the mutated sequence motif UCUU, UUCU, or CUCU in M1, as also observed in similar cases by Cao et al. (81). The full 68-nt probe sequence is described in Materials and Methods.

Upon closer examination of the RT-PCR products of the hormone genes under longer exposure and higher contrast, we consistently observed an extra band above the expected *Prl* in the shL samples only (Fig. 3A, lane 5). In lighter gels, this product is about 3% (molar ratio) of the *Prl* transcript. Importantly, the product was eliminated by hnRNP L-Flag expression in the shL cells (lane 6), which confirmed its control by hnRNP L. In contrast, the extra band was detected neither in the shLL samples, even with 8 times the *Prl* product loaded in the same gels (Fig. 1D, lane 6), nor in the shLL overexpressed samples (Fig. 1D, lane 7), suggesting that the band was caused by the loss of hnRNP L regardless of the presence or absence of LL. Moreover, no such extra bands were observed for the *Gh1* gene in any of these samples. Thus, hnRNP L but not LL is specifically required to prevent the presence of the longer *Prl* product in the endogenous transcripts.

Sequencing of the longer *Prl* product identified an intron piece of 93 nucleotides (nt) retained between exons 4 and 5 (Fig. 3B). It is flanked in the genomic DNA by the consensus dinucleotides AG and GT of splice sites but with an A at the first nucleotide in the exon and AAG/GTATTA at the 5′ splice site, both weaker than constitutive splice sites AG/G and AAG/GTAAGT according to their MaxEnt scores (8.28 and 7.04 versus 8.8 and 11, respectively) (38). This exon was not found in any of the about 250 mRNA/expressed sequence tags in the rat database of the UCSC Genome Browser (39), neither in the human or other mammalian species in the NCBI genomic databases by BLAST search nor by manual examination of the corresponding intronic regions. Moreover, this product was not seen in the RT-PCR assays of 14 rat tissues (unpublished data). Therefore, the 93-nt intron piece appears to be a rat-specific but cryptic exon upon hnRNP L knockdown.

The in-frame translated peptide from the exon is rich in basic residues (6 lysines and 1 arginine) (Fig. 2C) within the receptor-binding site of PRL, based on modeling of the variant structure using SWISS-MODEL and the crystal structures of the orthologous PRL-receptor complexes (40, 41). The location of the peptide therefore suggests an altered interaction of the aberrant PRL with the receptor and its function.

In addition to the longer *Prl*, there were also at least two shorter products in the hnRNP L-Flag-expressing sample (Fig. 3A, lane 6). They are from transcripts lacking exon 2 or exons 2 and 3 according to our sequencing data (Fig. 3B). These aberrant splicing events generate premature termination codons (PTCs) that likely cause nonsense-mediated mRNA decay (NMD) of the transcripts based on the rules of NMD (42–44).

Thus, different levels of hnRNP L appears to have an effect on the aberrant splicing of *Prl* in three directions: (i) its downregulation induces cryptic exon usage, accompanied by reduced normal *Prl* mRNA; (ii) its overexpression (at least 2-fold here) causes exon skipping and likely NMD; and (iii) only the endogenous level of hnRNP L is optimal for efficient proper splicing and expression of *Prl*. Although the hnRNP L-downregulation-induced cryptic product is not the majority of *Prl* transcripts and its role in the overall reduction of Prl is unknown, its presence suggests a novel role of hnRNP L to inhibit cryptic exons of endogenous transcripts in cells. Thus, we further examined this phenomenon.

The 93-nt cryptic exon of *Prl* and its flanking intronic regions contain several CA-rich sequences (Fig. 3D to F). Their role in the cryptic splicing was assessed in rat minigenes *Prl-1* to *Prl-3*, containing the 93-nt and different lengths of its upstream intron (Fig. 3D), in the vector DUP175 derived from the human beta globin gene (45, 46). RT-PCR analysis of their transiently expressed transcripts did not detect any cryptic variant in HEK293T cells, suggesting that the shortest transcript of *Prl-3* contains a sequence sufficient for the 93 nt to be inhibited (Fig. 3E). Further tests in shL- or shLL-expressing cells indicated that shL but not shLL allowed usage of the 93 nt (lanes 4 to 8). Importantly, the shL effect was eliminated by hnRNP L-Flag expression (lane 10). Thus, hnRNP L specifically inhibits usage of the 93-nt exon through sequences within the *Prl-3* transcript.

To identify the CA-rich sequences within *Prl-3* essential for hnRNP L binding, we carried out UV-cross-linking and immunoprecipitation assays of [α-^32^P]CTP-labeled RNA probes containing the 3 CA-rich sequence motifs or their mutants in HeLa nuclear extracts (Fig. 3F). The wild-type (WT) probe mainly cross-linked to a protein of about 65 kDa (lanes 1 and 2), which was eliminated by A-to-U mutations of all three motifs (M1; lane 3) but not by those in the first and last ones only (M2; lane 4). Moreover, the 65-kDa protein, but not the proteins between 35 and 48 kDa, was immunoprecipitated by the hnRNP L-specific antibody 4D11 (lane 5). Thus, the unmutated ACACAUCA and CACACA motifs are critical and sufficient for hnRNP L binding to the RNA transcript.

### hnRNP L specifically inhibits CA-rich cryptic exons in the transcriptome.

**FIG 4:**
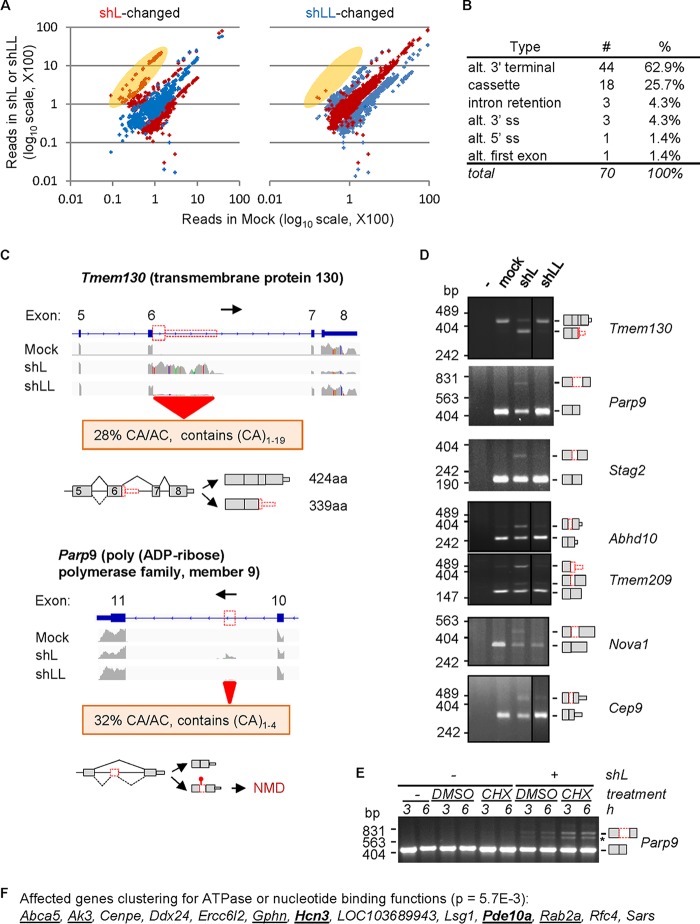
Transcriptome-wide analysis of intronic reads changed by shL or shLL. (A) Dot plots showing the number of reads of the 100-bp bins in shL (red) or shLL (blue) versus that in mock-treated cells. The yellow oval highlights bins upregulated at least 4-fold by shL/M. For comparison, the corresponding reads of bins in the shLL (left, changed by shL) or shL (right, changed by shLL) samples are also shown in each panel. (B) Types of alternative splicing represented by 416 shL-upregulated bins with ≥4-fold changes. ss, splice site. (C) Representative IGV views of the profiles of the RNA-Seq reads mapped to the predicted regions specifically in shL samples by edgeR intronic analysis. (Upper) Cryptic last exon of *TMEM130* gene. (Lower) Cryptic cassette exon of *Parp9* gene. Red boxes, the intronic cryptic exons; arrows, gene direction. The consequences of usage of the intronic sequences are at the bottom of each panel. The narrower boxes are 3′ UTRs. (D) RT-PCR validation of the predicted intronic RNA-Seq reads. Shown are agarose gels of RT-PCR products of seven genes tested (representative of at least 3 gels per gene with *P* < 0.001 for all except *Stag2* [*P* < 0.01]). −, PCR negative control. To the left of the gels are the sizes of DNA markers (in base pairs), and to the right are the splicing patterns of the cryptic exons (red boxes) and gene names. (E) Effect of cycloheximide (100 μg/ml) on the cryptic NMD exon of *Parp9* upon knockdown of hnRNP L by shL. *, product likely from an alternative cryptic splice site in the same exon. (F) DAVID functional annotation clustering of the affected genes for ATPase or nucleotide-binding functions. The genes coding for membrane or signaling proteins are underlined, and those critical for PRL release are in boldface.

To determine whether exclusion of cryptic exons, as in *Prl*, is a specific global effect of hnRNP L, we analyzed the RNA-Seq reads mapped only to introns of the Ensembl-annotated rat genome using edgeR. These introns did not have known annotated mRNA (exon) sequences in the assembly. We started with a stringent criteria by arbitrarily selecting bins (100 bp/bin) with more than 50 reads on average (among the three groups) and at least 2-fold changes over the mock samples. This resulted in 892 and 1,166 bins for shL and shLL samples (false discovery rate [FDR] of <5%), respectively, with only 34 (<5%) changed by both (Fig. 4A). Thus, their intronic target bins mostly (∼95%) do not overlap, again consistent with their mainly different target profiles of mRNA transcripts.

Most of the bins (>90%) are changed over values for the mock group by 2- to 4-fold. The downregulated ones could be rat exons still to be annotated in the database, and the upregulated ones could be either unannotated or cryptic exons. Interestingly, a distinct cluster of 78 bins, including the Prl 93-nt exon, showed more than 4-fold higher reads in shL than in the mock group (FDR of <2.4E−30) (Fig. 4A, left, yellow oval), with an average read of mostly less than 100 in the mock group. In contrast, no such bins were found in the shLL samples (Fig. 4A, right, yellow oval; the only 3 inside being from the shL sample). Thus, like its effect on the 93-nt *Prl* cryptic exon, hnRNP L knockdown appears to specifically enhance the usage of a group of cryptic exons that are barely spliced into mRNA in GH_3_ cells.

By manually analyzing all bins with at least 4-fold increases (FDR of <1E−4) in IGV, we identified 335 bins (81%) associated with the canonical GT-AG splice sites. Most of them (96%) have fewer than 100 reads in the mock group. They belong to 70 splicing events of 64 genes (Fig. 4B and Table S3), of which 62.9% are alternative last and 25.7% cassette exons. Thus, the majority of them would cause early polyadenylation and shortening of the mRNA transcripts, as observed for U1 snRNP and in cancer or other cells (47–50). In contrast, in the shLL group we identified only 92 such bins and only 7 such splicing events of 7 genes. Therefore, the increased usage of cryptic exons is mainly caused by shL.

The two major types of shL-targeting cryptic exons are exemplified in the IGV views of the last exon of *TMEM130* and the cassette exon of *Parp9* genes (Fig. 4C). Reads of these CA-rich pieces are barely detectable in the mock and shLL samples but clearly increased in shL samples. Their inclusion in the mRNA causes 85-amino-acid (aa) truncation at the COOH terminus of the protein (TMEM130) or a premature stop codon in the transcript (*Parp9*). Of seven such cryptic exons examined, we confirmed all of their specific increases in the shL samples by RT-PCR (Fig. 4D), as well as the further increase of the *Parp9* exon upon treatment by cycloheximide, an inhibitor of protein synthesis and NMD (51).

The CA/AC dinucleotides of these exons comprise about 30% of their sequences on average (*n* = 25 exons analyzed) (Table S3). Manual examination of 24 cases (6 alternative 3′-end and 18 cassette exons) did not identify any of them in the corresponding intronic regions of human or mouse genes. Moreover, in the above-described 14 rat tissues, none of the 4 examined exons was detected by RT-PCR (unpublished data). Furthermore, nine of the 18 (50%) cassette exons introduce premature termination codons. Consistently, higher usage of such cryptic exons (15%, on average, versus 5%; *P* value of 1.6E−06) is correlated with a 2-fold reduction of transcript levels (*P* value of 2.1E−05 using edgeR; *n* = 5) in shL samples. Therefore, these are intronic CA-rich, nonconserved sequences whose usage likely truncates transcripts/proteins or destabilizes mRNA transcripts.

A group of 13 of the 64 genes cluster most significantly for ATPase activities or nucleotide binding in DAVID functional analysis (*P* value of 5.7E−3) (Fig. 4F). Some of them, including *Pde10a* (phosphodiesterase 10A) (52), *Rab2a* (Ras-related protein Rab-2A) (53), and *Hcn3* (hyperpolarization activated cyclic nucleotide gated potassium channel 3) (54), are involved in cell signaling, vesicle formation, and electrical firing, which are important for hormone production or regulation. In particular, PDE10a, which hydrolyzes cyclic AMP (cAMP) (52), and HCN channels, which were found on vesicles, are critical for prolactin release (55, 56).

### The evolutionarily diverged proline-rich region (PRR) of vertebrate hnRNP L is essential for the exclusion of cryptic exons of specific genes.

**FIG 5:**
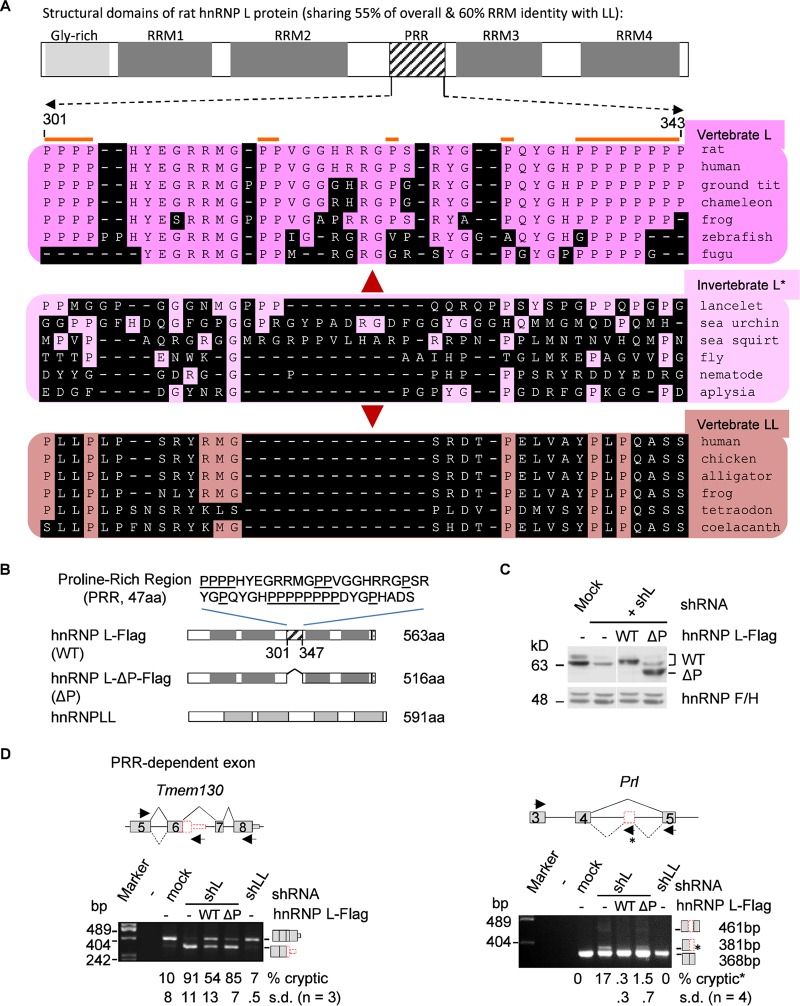
Comparison of the evolutionarily diverged PRR of rat hnRNP L with the same region of L and LL of other representative species (A) and the role of PRR in preventing aberrant splicing by hnRNP L (B to D). (A) Location of the PRR in the structural domains of rat hnRNP L and the aligned corresponding regions of different species using Clustal W. The diagram is drawn to scale. Amino acids different from the rat ones are shaded. *, invertebrate proteins have significantly higher percent identity to vertebrate hnRNP L than LL (39% versus 35%, *P* < 0.001, *n* > 40 pairs of proteins), using the same species as in reference 15; therefore, they are indicated as hnRNP L. The amino acid numbers are according to rat GenBank accession number EDM07871, as in the hnRNP L-Flag in this study. (B) Domain differences between the exogenous proteins of wild hnRNP L-Flag, hnRNP L-Flag with PRR deletion, and Myc-hnRNP LL. Above the domains is the deleted sequence of the PRR with prolines (P) underlined. Dotted box, Flag tag. (C) Western blots showing expression of the hnRNP L-WT-Flag (WT) and PRR deletion mutant L-ΔP-Flag (ΔP) in shL-transduced GH_3_ cells. (D) Representative examples of the cryptic exons in shL cells coexpressing WT or ΔP with a significant difference between the two hnRNP L proteins. Shown are splicing modes around the cryptic exons (upper) and agarose gels of the RT-PCR products (lower), with the inclusion level of the cryptic exons below each lane. Lines, introns; gray boxes, exons; red boxes, cryptic exons; arrows, PCR primers. Splicing modes are depicted with solid (normal) or dotted (cryptic) diagonal lines. Diagrams of the spliced products are to the right of the gels. The *Prl* cryptic product was amplified with an additional primer (star) in the 93-nt exon. The product from this primer was counted as the cryptic product.

We next investigated further the role of the diverged properties of hnRNP L and LL in preventing cryptic exon usage. Of their various domains (Fig. 5A), the RNA recognition motifs (RRMs) share about 60% sequence identity in Clustal W alignments. They also both harbor an N-terminal glycine-rich region. The only known domain that is in hnRNP L but not LL is the PRR, which has diverged evolutionarily into a distinct domain in vertebrate L from the corresponding region of its invertebrate ortholog. Interestingly, the PRR mediates protein-protein interaction specifically between hnRNP L and PTBP1 in preventing enhanced usage of alternative exon P3A of the acetylcholine receptor gene *CHRNA1* (57). We thus examined the effects of wild-type hnRNP L-Flag (WT) or its PRR deletion mutant (ΔP) on the cryptic exons in GH_3_ cells knocked down of hnRNP L (Fig. 5B and C).

Figure 5D shows the typical effects of PRR dependence of eight tested targets. Knockdown of hnRNP L, but not LL, significantly increased usage of both cryptic exons of *Tmem130* and Prl. The increase was partially reduced (*Tmem130*) or almost eliminated (*Prl*) by WT expression. The WT effect was either lost (*Tmem130*) or partially kept (*Prl*) upon deletion of the PRR domain. Partial reduction of the WT effects by the deletion was also observed for two other cryptic exons (*Abhd3* and *Abhd10*; *P* value of <0.05) but not significantly for the other four (*Nova1*, *Pla2g6*, *Cep19*, and *Tmem209*) (unpublished data). Therefore, the diverged PRR domain of hnRNP L is required for it to efficiently inhibit cryptic exons of specific genes.

### The remaining hnRNP LL paralog enhances the usage of cryptic exons in hnRNP L knockdown cells.

**FIG 6:**
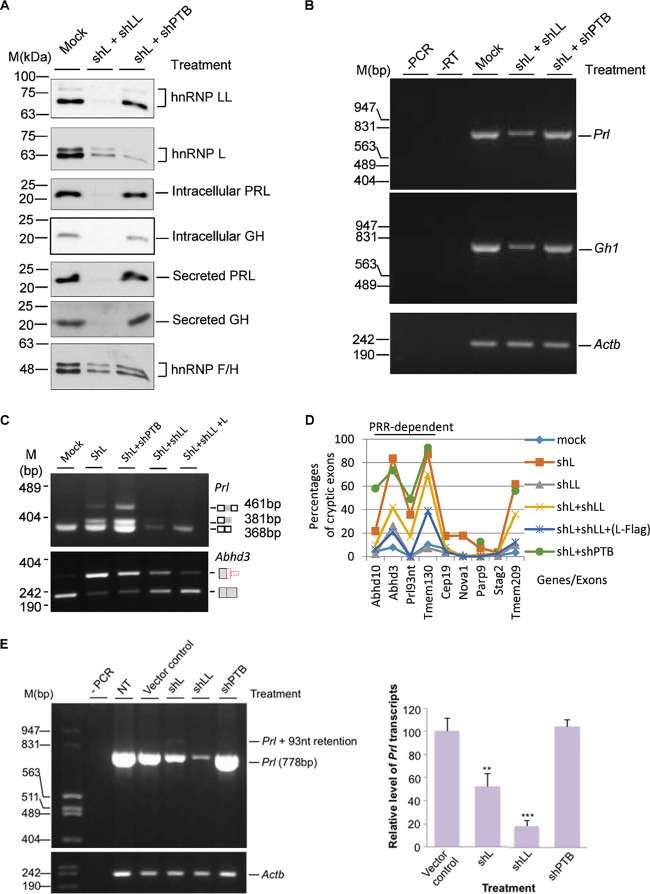
Effects of double knockdown of hnRNP L and LL or its known PRR-binding partner hnRNP I (PTBP1) on hormone production and the usage of cryptic exons. (A) Western blots of the splicing factors and hormones in GH_3_ cells with double knockdown of hnRNP L and LL or L and PTBP1 (shPTB). hnRNP F/H, protein loading control. Mock, mock transduced. (B) Agarose gels of the RT-PCR products of *Gh1* and *Prl* transcripts in the double knockdown cells. (C) Agarose gels of RT-PCR products of *Prl* and *Adh3* transcripts in single or double knockdown GH_3_ cells. Note that the Prl level is low in shL+shLL samples; therefore, to make sure the Prl93nt band is detected, a third (reverse) primer binding to the end of the 93-nt cryptic exon was included at a ratio of 10:1 to the downstream reverse primer. (D) Effect of double knockdowns of hnRNP L and LL or hnRNP L and PTBP1 on all the tested cryptic exons. (E, left) agarose gels of RT-PCR products of the *Prl* transcript in GH_3_ cells upon knockdown of PTBP1 (shPTB) compared with that of hnRNP L or LL. To the left are DNA size markers. (Right) Bar graph of the relative percentage of the *Prl* transcript. Data are means ± standard deviations; *n* = 3; **, *P* < 0.01; ***, *P* < 0.001. −PCR or −RT, PCR or RT negative control; NT, nontreated GH_3_ cells.

In shL or shLL knockdown cells, the other paralog had about 70% (LL) or 30% (L) increase, likely due to their cross-regulation, as reported previously (58). The hnRNP LL did not prevent cryptic exon usage upon hnRNP L knockdown (Fig. 4 and 5). However, whether hnRNP LL had an enhancing effect on the cryptic exons remains unclear. We thus tested the cryptic exons in both hnRNP L and LL knockdown cells. The double knockdown reduced the hormone or their transcript levels similarly to the knockdown of hnRNP LL alone (Fig. 6A and B). More interestingly, the double knockdown did not abolish the cryptic exons; however, it consistently reduced the usage of all nine tested exons compared to the corresponding shL samples (*P* value of <0.005 by paired *t* test; *n* = 9) (Fig. 6C and D). We also examined these exons in the double knockdown of hnRNP L and PTBP1, a binding partner of hnRNP L through the PRR domain (57), using the same lentiviral shRNA as in our previous work (30). This double knockdown did not have a consistent pattern of effects on the nine cryptic exons (Fig. 6C). Together, these indicate that the cryptic exons are inhibited by hnRNP L, and the remaining (increased) hnRNP LL in the shL-expressing cells likely had an enhancing, instead of inhibiting, effect on the cryptic exon usage.

Taken together, our data indicate that hnRNP L and LL differentially control hormone gene expression programs transcriptome-wide at at least three levels: total transcript, alternative exon, and beyond these normal targets of gene regulation, (inhibiting) cryptic exons within introns.

## DISCUSSION

Of the three levels of the differential control of hormone gene expression programs examined here in the pituitary cells, the regulation of total transcript level by hnRNP L and LL is likely through their control of mRNA stability, as was found in other reports (19, 59). Their regulation of splicing is well known, as explained in the Introduction. We thus have focused more on the third level: the hnRNP L inhibition of intronic sequences from aberrant splicing seen in this report. These effects together may play important roles in not only hormone gene expression but also transcriptome integrity and endocrine functions.

### Protection of the transcriptome from cryptic exons in introns by the expanded family of vertebrate hnRNP proteins.

Vertebrate introns have expanded and contain numerous cryptic exons that could endanger the integrity of many transcripts, but the relationship between transcriptome integrity and hnRNP expansion is not clear. Here, we have found a novel specific function of hnRNP L in protecting transcriptome integrity by excluding cryptic exons from mRNA. This effect requires its evolutionarily diverged domains for specific genes (Fig. 5), suggesting that the divergence of the hnRNP paralogs is necessary for the maintenance of transcriptome integrity besides the differential regulation of gene expression.

The difference in the intron targets of the hnRNP L and LL paralogs appears to involve intron sequence features, the diverged PRR, as well as other domains, including the RRMs. We detected hnRNP L but not LL binding to the *Prl* cryptic exon in the HeLa nuclear extract (Fig. 3E and F), consistent with their differences in preferred target motifs, as reported previously (17). However, the hnRNP LL upon hnRNP L knockdown appears to be required to fully promote the cryptic exons, since the double knockdown reduced these exons (Fig. 6C and D). One explanation for this could be that the hnRNP LL interacts with the target CA-rich motifs when hnRNP L is reduced, but it is not as repressive as when hnRNP L occupies the sites. In another case, this difference is due to the PRR domain of hnRNP L specifically mediating its interaction with PTBP1, whereas hnRNP LL cannot (57, 60), to repress 3′ splice site usage (57). The PRR domain is also required for hnRNP L to efficiently exclude specific, though not all, cryptic exons (Fig. 5), but double knockdown of the PRR-binding PTBP1 did not have a consistent effect on all cryptic exons (Fig. 6C and D). Therefore, other PRR-binding repressors might also be in play to inhibit the cryptic exons with hnRNP L.

The several PRR-independent cryptic exons appear to be mostly used at much lower levels than the ones dependent upon hnRNP L knockdown, except *Tmem209* (Fig. 6D), suggesting that at least some of their responses to the PRR deletion are sensitive to detection variations and thus are less likely to be significantly different between the wild type and PRR deletion mutant. We also examined their CA/AC contents, splicing type (cassette or last exons), and potential PTBP1-binding motifs nearby the CA-rich motifs (found in *Prl*, *Nova1*, *Cep19*, and *Pla2g6b*) (see Table S3 in the supplemental material) and found mostly no correlation with their PRR dependence or independence, except for the *Prl* exon. The inhibition of the PRR-dependent *Prl* cryptic exon by hnRNP L could be due to its PRR domain interaction with PTBP1, but the inhibition of these cryptic exons by hnRNP L and their PRR dependence or independence would need further investigation in regard to other features of hnRNP L unique domains (e.g., the NH_2_-terminal Gly-rich motif), other hnRNP L partners, more sequences, and preferably more cryptic exon targets.

Besides the hnRNP L and LL proteins, we also observed differential effects of hnRNP A1 and its paralog, A2B1, in inhibiting cryptic exon sequences enriched in GGG, triplets in the consensus motif of hnRNP A1 targets (61), from inclusion in the mRNA (unpublished data) by analyzing the intronic RNA-Seq reads of shRNA knockdown samples based on the raw sequences deposited in the NCBI SRA database by Geissler and colleagues (62). Moreover, hnRNP C inhibits Alu repeats and PTBP1 and PTBP2 inhibit CU-rich sequences in the introns (7, 8), although their paralog differences on the targets remain unclear. hnRNP LL also has a similar effect on intron retention (63). Such an inhibitory effect of hnRNP proteins on cryptic intronic sequences has also been supported by other studies but more commonly in splicing reporters, for example, by hnRNP A1 (64). Together, the current pieces of evidence suggest that at least a group of hnRNP proteins have diverged to differentially protect transcriptome integrity beyond their roles in the regulation of gene expression. The expanded different proteins, including paralogs, thus may provide diverse *trans*-acting factors to recognize the different sequence motifs (15, 65–67) to guarantee efficient inhibition of cryptic exons inside long introns. We anticipate that more diverged hnRNPs or their paralogs with different sequence preferences will be identified to inhibit transcriptome-wide cryptic exons in vertebrate cells as more and more cases of intron read mapping are applied in future studies.

### The multilevel control of genes by different hnRNPs in endocrine functions.

The transcript level appears to be the major control for growth hormone and prolactin by both hnRNP L and LL. Overall, the genes regulated at this level clustered differentially for cation transport and guanyl nucleotide binding by the paralogs. At the exon level, the target genes also clustered differentially, for transcription factors and signaling by the hormone oxytocin (hnRNP L), and for cytoskeleton and nucleotide binding (hnRNP LL). Inhibition of cryptic exons is mainly an effect by hnRNP L in these cells; this effect is critical for the proper expression of many hormone- or hormone-related genes, including the prolactin (Fig. 3 and 4). Therefore, the multilevel differential control of hormone gene expression programs by hnRNP L and LL covered ranges of molecular processes of endocrine functions involving at least growth hormone and prolactin production, cell signaling, excitability, vesicle transport, and gene transcription, as well as the production of other hormones (Fig. 2 and 4). This differential control may serve to tailor and safeguard the transcriptome for specific endocrine functions. It would be interesting to investigate whether this differential control also plays a role in the production of distinct hormones during the differentiation of the common precursor pituitary endocrine cells into lactotrophes and somatotrophes in the pituitary (32).

In summary, our findings demonstrate that the hnRNP L and LL proteins differentially control gene expression programs at multiple levels in the production of two physiologically important hormones. Particularly interesting is their differential roles in globally inhibiting the inclusion of cryptic intronic sequences in mRNA to protect transcriptome integrity. The multilevel differential control by hnRNP proteins likely contributes to the fine-tuning and protection of the gene expression programs and cell physiology. These effects again emphasize the importance of investigating hnRNP deregulation and aberrant processing of RNA transcripts, particularly intronic sequences (RNA-Seq reads), in diseases (68–70).

## MATERIALS AND METHODS

### Plasmid construction.

The lentiviral plasmids pFG12-shL (shL) and pLKO.1-shLL (shLL), against hnRNP L and hnRNP LL, respectively, and pCppt2E-hnRNP L-Flag have been described previously (28, 29). To express hnRNP L-ΔP-Flag, the PRR-encoding fragment was deleted by PCR from hnRNP L-Flag and subcloned into pCppt2E, as described by Yu et al. (28). Human hnRNP LL cDNA (with an open reading frame from GenBank accession number NM_138394.3; Open Biosystems) was cloned into pCMV-Myc (29) and then subcloned into pCppt2E (29).

A silent mutant of pCppt2E-Myc-hnRNP LL (T342A, G348T, and A351G) was generated by site-directed mutagenesis for rescue experiments based on Fisher's procedure (71). Briefly, a 25-μl mutagenesis PCR was carried out with 50 ng of cppt2E-Myc-hnRNP LL plasmid, complementary mutant primers, and 2 U of Phusion high-fidelity DNA polymerase (New England BioLabs, Inc.) for 18 cycles (95°C for 60 s, 55°C for 60 s, and 68°C for 20 min [2 min per kilobase of sequence]). The PCR product was digested with 10 U of DpnI at 37°C for 1 h and transformed into Escherichia coli DH5α.

For *Prl* splicing reporter minigene *Prl-1*, a genomic DNA fragment of the 93-nt cryptic exon with partial flanking introns (829 bp upstream and 374 bp downstream) was amplified using primers PRL-WT5 (5′-CGGGCCCTGCTTTCTGCAATGAGGAAC-3′) and PRL-WT3 (5′-GAGTTGTGACCAAACCAAGTAG-3′) from rat genomic DNA, digested with ApaI/BglII, and inserted into the DUP175 splicing vector (46). Further deletions (*Prl-2* or *Prl-3*) and mutations were made by PCR based on this template.

All resulting plasmids were confirmed by sequencing.

### Cell culture and transfection.

GH_3_ cells were maintained in Ham's F10 nutrient mixture with 10% horse serum (HS), 2.5% fetal bovine serum (FBS), and 1% penicillin-streptomycin-glutamine solution (PSG). HEK293T human embryonic kidney cells were cultured in Dulbecco's modified Eagle medium (DMEM) supplemented with 10% newborn calf serum (NCS) and 1% PSG. For minigene assays, the reporter plasmids were transfected with Lipofectamine 3000 (Invitrogen) according to the manufacturer's protocol.

### Lentivirus transduction.

Preparation of lentiviral particles for shRNA, hnRNP L-Flag, L-PRR deletion mutant (ΔP), or Myc-hnRNP LL expression and lentiviral transduction was done as described previously (28, 29).

### RT-PCR.

Cytoplasmic RNA was fractionated and extracted from GH_3_ cells with or without lentivirus transduction according to our previous procedures (28, 72). About 400 ng of cytoplasmic RNA was used for 10 μl of reverse transcription reaction mixture. PCR for gene expression and splicing analysis was performed for 28 to 31 cycles for different genes (28 cycles for *Actb* and *Gh1*, 30 cycles for *Prl*, and 31 cycles for *Prl* splicing analysis). PCR products were resolved in 1.5 to 3.5% agarose gels containing ethidium bromide (EtBr) and visualized with a digital camera under UV. The percentages of splice variants were calculated from band intensities unless otherwise stated.

GH_3_ total RNA for RNA-Seq was extracted using an RNeasy Plus minikit (Qiagen) by following its protocol. Similar RT-PCR procedures were followed for validation, except about 500 ng of total RNA was used for 10 μl of reverse transcription reaction mix.

### Western blot analysis.

Western blot analysis was performed according to reference 73, with slight modifications. For cell lysates, approximately 10 μg of total protein or 5 μg of nuclear protein was resolved in 10% SDS-PAGE. For secreted hormones, 7.5 μl of fresh medium (12 to 24 h after change) was resolved in 12% SDS-PAGE. Proteins were transferred overnight to polyvinylidene difluoride (PVDF) membranes at 100 mA at 4°C, blocked with 5% fat-free milk for 1 h at room temperature, incubated overnight with primary antibody at 4°C, and incubated with secondary antibody at room temperature for 1 h. Proteins were detected by applying ECL Western blotting detection reagent and exposed to X-ray films. Anti-hnRNP L (4D11) and anti-hnRNP F/H (1G11) were purchased from Santa Cruz Biotechnology Co., anti-hnRNP LL (4783) from Cell Signaling Technologies, anti-Flag (M2; F1804) from Sigma-Aldrich, anti-PRL (6F11) from Pierce Co., and anti-GH from the National Hormone and Peptide Program (NHPP; Harbor-UCLA Medical Center).

For equal loading of culture media, Ponceau S staining after protein transfer and hnRNP F/H levels of the same samples were monitored in experiments.

The index levels of the hormones were normalized to the loading and transduction controls.

### UV cross-linking and immunoprecipitation.

The assay was performed with [α-^32^P]CTP-labeled RNA probes and HeLa nuclear extracts as described previously (28). RNA probes were *in vitro* transcribed using T7 RNA polymerase from DNA templates (5′-TGTATGTGTGTTTAATCTGATGTGTACAGTTGTctgaaaattacaaaaatatagcatgcatttgcataccctatagtgagtcgtatta-3′, WT, with the reverse complementary sequence of the T7 promoter underlined, uppercase indicating the exon, and lowercase indicating the intron except for the T7) preannealed with equal molar amounts of T7 promoter oligonucleotide. The final WT RNA transcript sequence is uaugcaaaugcaugcuauauuuuuguaauuuucag**ACAAC**UGU**ACACA**U**CA**GAUUAA**ACACACA**U**ACA**, with the CA/AC clusters in boldface. Anti-hnRNP L (4D11) was used for immunoprecipitation.

### RNA-Seq and bioinformatics analyses.

Approximately 3 μg of total RNA from samples of the three groups, mock (nontransduced; treated with 0.8 ng/μl Polybrene), shL, and shLL (samples in triplicate in each group), were reverse transcribed using oligo(dT) for cDNA library construction and Illumina Hi-Seq2000 sequencing. RNA quality control, library preparation, and Illumina Hi-Seq 2000 paired-end 100-bp sequencing were conducted at the McGill University and Génome Québec Innovation Centre (Montréal, Québec, Canada).

An average of 189 ± 42 million paired reads were mapped to genome assembly Rnor_5.0 for 15,169 ± 247 genes per group. The differential gene expression and aberrant intronic splicing were calculated in the Bioconductor package edgeR (74, 75). Alternative exon usage was calculated in the package DEXSeq (37), with a validated filter developed in our laboratory. Basically, we filtered out those bins with exonbase means and average exonbase means of less than 50, with a ratio to the highest exonbase mean (after normalized to bin length) of less than 0.002. To visualize the mapped reads of genes/exons, we used the Integrative Genomics Viewer (IGV; developed at the Broad Institute of MIT and Harvard; http://www.broadinstitute.org/igv) (76). The cryptic exons were visually examined in IGV, with the cassette exons characterized by the steep drop of read peaks accompanying the splice site dinucleotides GT/AG and the alternative 3′-end exons by the long length (mostly >300 nt), with read peaks slowly declining to zero or baseline at exon ends, no obvious downstream 5′SS GT dinucleotides, and the presence of AAUAAA or AUUAAA motifs (77, 78).

For functional annotation and classification of genes, we used the Database for Annotation, Visualization, and Integrated Discovery (DAVID; developed at the U.S. National Institute of Allergy and Infectious Diseases; http://david.abcc.ncifcrf.gov) (79).

### Image data analysis.

Images were quantified with the ImageJ software (developed at the U.S. National Institutes of Health; http://rsb.info.nih.gov/ij/) (80).

### Statistical analysis.

Data are presented as means ± standard errors of the means for at least three independent experiments. Statistical analysis was done with two-tailed unpaired Student's *t* test, except for the built-in tests in DAVID (modified Fisher's exact test) or Bioconductor packages.

## Supplementary Material

Supplemental material
