# Supplementary material for: Multilevel Differential Control of Hormone Gene Expression Programs by hnRNP L and LL in Pituitary Cells
Source: Mol Cell Biol. 2018 May 29;38(12):e00651-17. doi: 10.1128/MCB.00651-17 (PMC5974433; doi:10.1128/MCB.00651-17)
Supplement: Supplemental material [file supp_38_12_e00651-17__index.html]

Supplemental material 

# Multilevel Differential Control of Hormone Gene Expression Programs by hnRNP L and LL in Pituitary Cells

## Supplemental material

- Supplemental file 1 -

  Table S1 (DAVID functional annotation clustering analysis of genes whose expression levels are preferentially regulated by hnRNP L or hnRNP LL in edgeR analysis)

  XLSX, 143K
- Supplemental file 2 -

  Table S2 (Exons/regions that changed significantly between at least two pairs of three groups of 120 samples)

  XLSX, 407K
- Supplemental file 3 -

  Table S3 (List of intronic “exons” increased by shL)

  XLSX, 40K
